# Case of Diabetes Mellitus

**Published:** 1816-07

**Authors:** C. W. Smerdon

**Affiliations:** Member of the Royal College of Surgeons.


*For the London Medical and Physical Journal.*

### *Case of Diabetes Mellitus;* by Mr. C. W. Smerdon, Member of the Royal College of Surgeons.

W. WILSON, aged 45, of a thin spare make, small stature, long face, and light complexion, was attacked, about five years ago, with Diabetes Mellitus. He was butler in a respectable family in Yorkshire, and, at the commencement of his illness, had been for many days constantly employed in brewing, during which he drank freely of sweet-wort. Being naturally delicate in constitution, although he has never had any serious illness, his habits of living were always regular. According to his account, the diabetic symptoms were at this time strongly marked; but the one which more particularly attracted his attention was a guggling sensation, as if water or air was passing through tubes from the upper to the lower part of his bowels. To relieve the disordered state of his stomach, he took several emetics, which he thought were attended with beneficial effects. He was likewise strictly enjoined to persevere in an animal diet, and he came to this place for the benefit of the air and water.

By strict attention to this plan of treatment, and by drinking freely of our hot-well water, he gradually recovered his strength, his urine became natural as to quantity, and he returned convalescent to his former situation. He remained tolerably well for some months; but, in consequence of great fatigue, and exposure to cold and wet, was again attacked with with the disease, although he had steadily persevered in the animal diet. His appetite now became voracious, and his thirst and fever much more urgent than before. After trying a variety of remedies to no purpose, he returned to the Bristol hot-wells. I first saw him about two years after this time, during which he has been progressively, though slowly, getting worse.

Sept. 19, 1815.—Saw him, for the first time, this day. His whole appearance is strongly characteristic of premature old age. His sight is very much impaired; pupils sluggish and contracted; and his hair is nearly all white. His tongue is deeply furred in the centre, and covered with a layer of hardened mucus, which is surrounded by a red border. The texture of his skin, in consequence of long absence of perspiration, is apparently changed: it is hard, thick, and scaly, resembling goose-skin. His mouth and nostrils are always dry and uncomfortable, his sputum frothy, but not sweet. During the whole of his illness, he does not recollect having suffered any acute pain, but he complains of a sensation of weariness or lassitude, a feeling of illness, as he expresses it, all over him, without being able to particularize one part more than another. He has a constant craving for drink, which he refrains from gratifying, as much as possible, because he is convinced that the more liquid he takes the more thirsty he becomes. His best time is in the morning; but after having eaten a full meal, as at dinner, the wearisome feel and thirst are increased. Towards evening, he has regularly an attack of fever: this first commences in his loins, which feel particularly hot and unpleasant; he becomes restless and uneasy, and the greater part of the night is thus spent without sleep. The palms of his hands are red and dry, and his feet are swelled at the instep. He makes, on an average, about nine pints of straw-coloured urine in the twenty-four hours: this has a fragrant vinous smell, and sweet taste, and is evidently in a state of fermentation when evacuated. He has not kept an account of the quantity of liquid drank, but thinks it may amount to six or seven pints: at any rate he is positive (which no argument as to its improbability can divest him of) that he drinks much less than the quantity of urine evacuated. The retentive powers of his bladder are as good now as when he was in perfect health; nor has he any uneasiness, either before or after making water; he voids about a pint and a half at a time, at intervals of about four hours, and could retain it even longer than this, if necessary, without inconvenience. He has periodical discharges of blood by the urethra every two or three weeks; the quantity of blood lost each time is not

 more more than an ounce, and is not attended with any pain. The animal diet, for some time after he commenced it, continued to have the most beneficial effect; his urine, in consequence, lost its sweetness, was natural in quantity, and he gained strength: the slightest deviation, however, from a strict regimen, was always followed by a sweetness of the urine. At length the animal food gradually began to lose its influence, and at this time it is so far lost, that he takes toasted bread and tea for his breakfast, and the same in the evening; and he thinks that this change has lessened the fever and the thirst, whilst the quantity and quality of the urine are the same. About a week ago, he began to take opium, to the extent of six grains a-day. His pulse was then very feeble, at 120 in the minute. He has been much better since. The opium does not make him sleepy, but it lulls the unpleasant sensation of his loins, takes off the wearisome feel, and gives him strength. His pulse is now 100, full and strong. His bowels are mostly in a torpid state, and the evacuations *per anum* generally small in quantity; but the operation of aperient medicine disorders him very much, although it diminishes considerably the quantity of urine. He took lime-water in large doses daily for some weeks, and also the sulphuret of potash, without experiencing any benefit, whilst he is satisfied that the Bristol hot-well water had great influence in relieving his first attack. It is remarkable that, notwithstanding his whole appearance indicates extreme debility, he experiences little or no fatigue from walking three or four miles at a time.

Sept. 20th.—He was bled to 14 oz.; pulse full and strong, at 100 in the minute; made, last night, four pints of straw-coloured sweet urine; no stool yesterday; to take some cathartic pills.

23d.—He was pretty well during the day after the bleeding, but passed a restless night, in consequence of head-ach and nausea. On the following evening, the cathartic pills which he had taken operated by the bowels, and he spent a more composed night than he has done for some time before. He scarcely voided a pint of urine, which is more natural than any he has previously made; smell more urinous; taste a little bitter, but not perceptibly sweet. Pulse strong, at 120 in the minute; less thirst; skin cooler; serum of the blood taken on the 20th remarkable for being of a milk-like colour; taste not quite so saline as usual, but certainly not sweet. The crassamentum is firm, but not cupped or buffy. Bled again to 14 oz.; bore the operation. He is requested to eat moderately of a mixed diet, and to put a blister on his loins.

26th.

26th.—Has felt himself better since last report; has made on an average five pints of urine in twenty-four hours, which he is now certain is less than he drinks of liquid; skin cooler; less thirst; pulse moderately strong at 100 in the minute; nights more composed; and he has lost the unpleasant sensation in his loins. The blood last taken has a healthy appearance, is not cupped nor sizey; crassamentum could be suspended on the point of a knife; bled to 14 oz.

Oct. 1st.—Has continued better, in every respect, until yesterday afternoon: the worst symptoms of the disease, which before were mitigated, began then to return. He complains of excessive lassitude; his urine is greater in quantity, and sweeter, than at last report. Upon the whole, he thinks himself stronger since the bleeding, in which he still has great faith. Pulse 90, rather weak; bled to 12 oz.; bore the operation well; blood last taken healthy; sleeps better at night; skin cool, but no perspiration.

4th.—Made, last twenty-four hours, seven pints of straw-coloured urine; no deposition on standing; taste bitter, and rather sweet; feels weak; appetite delicate; loathes animal food; blood last taken healthy; crassamentum firm, but not cupped nor buffy; has lost no blood by the urethra since the commencement of the bleeding; is free from the unpleasant heat about his loins, but the guggling sensation in his bowels still continues unalleviated; bled to 8 oz.

6th.—No amendment; feels weak; urine still continues sweet, but less so than before the bleeding—the loss of sweetness is supplied by a bitter principle; averages seven pints a-day. Upon the whole he is much better now than before he began with the opium: this gives him strength, lulls the wearisome feel, and raises his spirits. The bleeding has allayed the fever, and his nights are more composed. Wishes to go to Bath for the purpose of trying warm bathing.

Nov. 1st.—He has been in Bath for the last three weeks, but no material benefit has resulted from the warm bathing; no disposition whatsoever to perspire; has been bled also five times, by which he lost 4½ lbs. of blood; he is much weaker now than when I last saw him; appetite not improved; made, in the last twenty-four hours, eight pints of straw-coloured urine; smell more natural than usual, and is less sweet; he is convinced that he voids more urine than he drinks of fluid; pulse 80, moderately strong; skin more natural than before the batning; bowels tolerably regular. —To take twenty drops of the Tinct. Lyttæ three times a-day, and ten grains of Dover's powder every night.

10th.—Appears to be sinking fast; has omitted the opium for the two last days, and consequently since then he has been been rapidly declining; debility and languor so great that he can scarcely walk across the floor; thirst much increased; is constantly craving for drink, and makes daily about thirteen pints of urine; feels as if every thing which he takes into his stomach passes off again by urine; pulse regular, but languid; is desired to resume his opium, and increase it to eight grains a-day; to omit the powders, as they make him feel hot and uncomfortable at night, and to augment the Tinct. Lyttæ.

13th.—No amendment; thirst and urine the same.

℞. Opii, gr. xxiv.
Calomel & Digitalis āā gr. iv. M. ft. Pilulæ No. ix. capt. i. ter. die—etiam capiat cum sing. Pilulus.
Pulv. Cinchonæ, ʒſs.

17th.—Disease appears to be checked; thirst moderated; urine about seven pints in the twenty-four hours.—Cont. remedia.

21st.—Is still better; countenance and strength much improved; increase the calomel and digitalis to 6 grains, and tinct. lyttæ to 40 drops.

It would be uninteresting to go on further in detail with this case. He remained better until the 27th, and then fell off again to his former state. At this time the tinct. lyttæ had been increased to 60 drops three times a-day, and I am deceived if it produced any sensible effect upon the urinary organs. Were the calomel and digitalis instrumental in bringing about the last temporary suspension of the disease? On the 28th his mouth became sore, and for the first time discovered that he was taking a mercurial preparation, which had a very visible effect upon his spirits, and, I might add, upon the disease. His bowels having been confined, he has taken two doses of purging pills, which, although they did not operate much, have kept up a considerable irritation on the bowels. He wishes, for the present, to leave off all medicine but the opium. The bleeding from the urethra has returned the same as before. Since then, he has taken preparations of steel, the sulphuret of potash, soda, columba, and bals. copa. at different periods, along with the opium; but none of them, except the latter, has had the least influence either on his general health, or on the quality of the urine. The opium is his sheet-anchor. Without this valuable remedy, I am satisfied that his miserable existence must have terminated long ago. For some time past he has taken eleven grains and a half daily, and sometimes thirteen grains, when he has been unusually unwell. The disease, however, has now nearly arrived to its fatal close. About three weeks ago, he was attacked with a tickling cough, cough, attended by an olive-coloured mucous expectoration, which is now so distressing that he cannot talk without great difficulty, and his rest is disturbed by night. This cough is evidently the effect of irritation (from some cause or other) of the mucous membrane lining the trachea and bronchial ramifications. The powers of his stomach appear to have undergone a considerable change, for, instead of the usual sensation of emptiness, he now feels blown out, as he expresses it, after eating, and otherwise so very uncomfortable, that he refrains from taking food as much as he can, although his appetite is craving. His urine is also decreased in quantity, from eight or nine pints a-day to four pints, the sweet taste of which is now scarcely perceptible, but it is a little bitter, and it has lost the vinous smell and effervescent quality. It is not, however, difficult to account for this change. The diabetic disease has at length so far impaired the powers of life, that the nice balance, so necessary to health, between the absorbent and the exhalent systems, appears to be lost. His legs and feet are distended to the utmost with fluid. He has water also in the abdomen, and (I suspect) in the chest. Such is the approaching termination of this extraordinary disease.

I must confess that I began the bleeding plan on this poor man with the most sanguine hopes of success; and therefore the trifling benefit which he derived from it was to me a source of much disappointment. I beg, however, that this remark may not be construed into a wish to undervalue that powerful remedy: on the contrary, it is my present intention to publish, in a future communication, the substance of another case, together with conclusions drawn from the writings of others, which will, in my opinion, give to bleeding in diabetes the degree of importance which it justly deserves.

*Distinction between Diabetic and Healthy Urine.*

If fresh healthy urine be evaporated by boiling to about two-thirds of the whole, a reddish-coloured deposition takes place, consisting chiefly of the phosphates of lime and magnesia. On treating the supernatant liquor with the solution of the muriate of lime, and suffering the mixture to stand, a copious orange-coloured precipitate is thrown down, which is rapidly dissolved on the addition of a little muriatic acid. On evaporating, in the same manner, the diabetic urine which I obtained, no deposition took place, but the liquid began to thicken. The oxalate of ammonia (the test for lime), the phosphates of soda and ammonia (the tests for magnesia), and the muriate of lime (the test for urea), when separately

 tried, tried, threw down no precipitate. I obtained crystallized urea by digesting in alcohol some healthy urine, which had previously been evaporated to the consistence of syrup, by allowing the deposit to settle, and again evaporating the clear liquid to the same consistency, and setting it aside that crystals might form. Urea, thus obtained, when the uncrystallized liquor is drained from it, is of a bright orange colour, a strong urinous smell, and ammoniacal taste. Diabetic urine, when evaporating, begins to thicken much sooner than healthy urine; the sides of the vessel soon become lined with a black crust, which, when brought to the consistence of syrup, is of a dark brown colour and sweet saline taste. If a small quantity of spirit of wine be poured on this mass, and the whole briskly stirred together, the clear liquor, when separated from the deposit by rest, contains some of the salts of the urine, the bitter principle, if the urine had been bitter, but none of the sugar. This liquor yields, on evaporation, a brownish saline extract, transparent, and similar in appearance to new honey. On mixing a little lime-water with the residuum which remained after the spirituous solution had been poured off, the lime was precipitated; and the supernatant liquor, when boiled down to a proper consistence, could not be distinguished from syrup which had been made with impure sugar. Rather copious precipitates were thrown down by the nitrate of silver, and the muriate of barytes.

These experiments naturally lead to the following conclusions:

1st. That diabetic urine contains neither urea nor the earthy phosphates.

2dly. That it contains a considerable quantity of a brown extract, united with a small proportion of sugar.

And 3dly. When this sugar is absent, its place is sometimes supplied by a bitter principle.

*Clifton, Bristol;*

*April 12th,* 1816.

